# Spatiotemporal Patterns of Suitable Wintering Habitats for the White-Naped Cranes Under Climate and Land-Use Change

**DOI:** 10.3390/ani16121839

**Published:** 2026-06-15

**Authors:** He Xiao, Mingqin Shao, Zeng Jiang

**Affiliations:** Key Laboratory of Biodiversity Conservation and Bioresource Utilization of Jiangxi Province, College of Life Sciences, Jiangxi Normal University, Nanchang 330022, China; xiaohe@jxnu.edu.cn (H.X.); jiangzeng@jxnu.edu.cn (Z.J.)

**Keywords:** White-naped Crane, MaxEnt model, suitable wintering areas, climate change, spatiotemporal variation

## Abstract

The White-naped Crane (*Antigone vipio*), a first-class national protected bird species in China, exhibits a declining global population. To investigate the spatiotemporal patterns and drivers of wintering habitat suitability, data from 71 valid distribution sites were collected from 2015 to 2025 during the wintering period. Using the MaxEnt model, current and future (2050 and 2070) potential suitable habitat distributions were simulated under three climate scenarios: SSP126 (low emissions), SSP245 (medium emissions), and SSP585 (high emissions). The modeling yielded an average AUC value of 0.984, indicating high predictive accuracy. Key environmental variables influencing the wintering distribution of the White-naped Cranes include elevation, distance to major water, precipitation of the driest month, slope, temperature seasonality, and mean temperature of the wettest quarter. The current high-suitable area for the White-naped Cranes spans 5.64 × 10^4^ km^2^ and is primarily distributed in the middle and lower reaches of the Yangtze River and in coastal wetlands along the North China. Among these, Hunan, Hubei, Jiangxi, and Anhui provinces contain relatively concentrated high-suitable areas for the species. Primarily influenced by elevation, distance to major water, precipitation of the driest month, and land-use classification, the suitable wintering habitat of the White-naped Cranes is projected to undergo significant contraction, shifting predominantly to the middle reaches of the Yangtze River. The most severe contraction is projected under the SSP585 scenario by 2070, with a reduction of 4.11 × 10^5^ km^2^. Contraction areas are primarily concentrated along the Bohai and Yellow Sea coasts and in the middle and lower reaches of the Yangtze River, while minimal expansion occurs in Hubei, Anhui, and Zhejiang. The overall southwestward shift in the species’ distribution centroid may be associated with changes in elevation and distance to major water. Finally, habitat conservation strategies for the White-naped Cranes are proposed, providing a scientific basis for population protection and habitat management under future climate change.

## Simple Summary

This study collected data from 71 distribution sites of wintering White-naped Cranes between 2015 and 2025. Using the MaxEnt model, we simulated the distribution of potential suitable habitats for the White-naped Cranes based on environmental variables such as elevation, climate, land-use classification, and human disturbance. The simulation periods cover the present and future (2050 and 2070), encompassing three climate scenarios: SSP126 (low emissions), SSP245 (medium emissions), and SSP585 (high emissions). The results indicate that the key environmental variables affecting wintering White-naped Crane (*Antigone vipio*) distribution include elevation, distance to major water, precipitation of the driest month, slope, temperature seasonality, and mean temperature of the wettest quarter. The current high-suitable areas for the White-naped Cranes is primarily distributed in the middle and lower reaches of the Yangtze River and in coastal wetlands along the North China. Under all future climate scenarios, the distribution centroid is projected to shift southwestward overall. The suitable habitat range is projected to primarily relocate to the middle Yangtze River region, with significant contraction in other areas. This shift may be associated with changes in future climate and land-use classification.

## 1. Introduction

The wintering patterns of waterbirds are primarily determined by factors such as topography [1,2], climate [3], water availability [4,5], and human disturbance [4,5]. Currently, bird populations worldwide are being significantly affected by climate change, and rising temperatures are expected to drive shifts in habitat distribution [6]. Under future greenhouse gas emission scenarios, suitable habitat for the Siberian Crane (*Leucogeranus leucogeranus*) and the Hooded Crane (*Grus monacha*) in the middle and lower Yangtze River is projected to decline markedly, with the distribution centroid shifting toward higher latitudes [6,7]. Similarly, the wintering range of the Black-necked Crane (*Grus nigricollis*) is also projected to contract, with its distribution centroid shifting toward higher-altitude regions in western areas [8]. Global warming is expected to alter land-use composition in wintering grounds, thereby prompting some crane species to adjust their foraging strategies and increasing their reliance on anthropogenic habitats [7].

The White-naped Crane (*Antigone vipio*) is classified within the Gruidae of the Gruiformes [9,10]. It is designated as a Class I nationally protected species in China and is classified as Vulnerable (VU) on the IUCN Red List [9,10]. It primarily overwinters in regions such as Poyang Lake, Dongting Lake, and Shengjin Lake [11], where marsh and grassland habitats adjacent to rivers and lakes are preferred, and its diet consists mainly of aquatic plants [10]. The global population is currently estimated to be approximately 7000–7800 individuals, with fewer than one-quarter wintering in China [12], and the overall population trend is declining [9,13,14]. Previous studies have addressed multiple aspects, including gut microbiota [15], migratory behavior [16], population dynamics [11], habitat quality [17], and species distribution [18]. However, the structure and function of wetland ecosystems along its migratory routes have been substantially altered [14]. The patterns of current and future shifts in its wintering distribution remain poorly understood, thereby hindering effective conservation and long-term habitat management. Species Distribution Models (SDMs) are grounded in the concept of ecological niches [1,7,19]. Potential suitable habitats of target species are predicted by analyzing relationships between occurrence records and environmental variables [1,7,19]. Currently, various types of distribution models are available; among these, the MaxEnt model is one of the most widely used due to its ability to perform well with limited occurrence data and its high predictive accuracy [1]. To date, the MaxEnt model has been extensively applied to predict current and future suitable habitats for waterbirds, with well-established methodologies and high predictive performance [1,2,3,4,5,6,7,8,20]. The application of the MaxEnt model to predict future distribution patterns of large rare species such as the White-naped Crane is considered essential for long-term conservation and habitat management planning [7,21]. Additionally, such predictions provide valuable information for species conservation, habitat restoration, and protected area planning [6,7,8].

In the context of global warming, suitable wintering habitat for the White-naped Crane is hypothesized to decline significantly, with two potential patterns: (1) under increased emission scenarios, habitat is projected to decline markedly in the middle and lower Yangtze River and southern regions, with moderate expansion in some northern areas; and (2) under a scenario of continued rising greenhouse gas emissions, habitat is projected to decline substantially in the coast wetland in North China due to the loss of water resources, potentially rendering these areas unsuitable for wintering, with only limited habitat remaining in parts of the middle and lower Yangtze River. To test these hypotheses, the MaxEnt model is applied to examine spatiotemporal changes in wintering habitat and their underlying drivers, with the following objectives: (1) to predict potential suitable wintering habitats under current and future climate scenarios; and (2) to identify key environmental variables influencing wintering distribution and to inform conservation strategies for population protection and habitat management.

## 2. Materials and Methods

### 2.1. Distribution Data of the White-Naped Cranes

**Figure 1:**
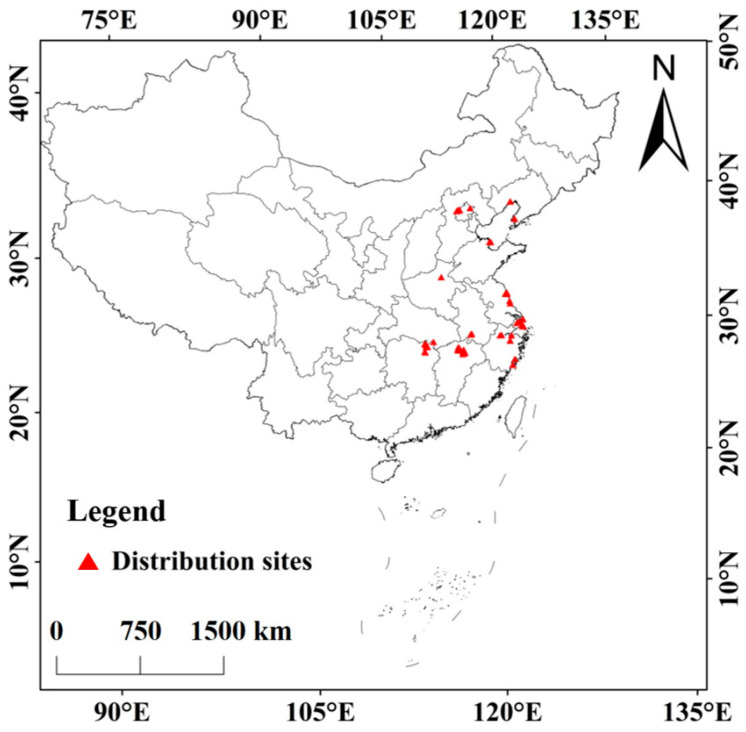
Wintering distribution map of the White-naped Cranes.

Distribution site data for the White-naped Cranes were primarily obtained from the China Birdwatching Records (http://www.birdreport.cn, accessed on 10 August 2025) and the GBIF Occurrence Download. Available online: http://www.gbif.org (accessed on 13 August 2025). A total of 94 raw records of wintering distribution locations across China were collected annually in January (the stable wintering period) from 2015 to 2025. The raw site data were imported into ArcGIS 10.8, and 1 km radius buffers were generated. Only one site per grid cell was retained to reduce sampling bias in the dataset. Ultimately, 71 valid sites were identified (Figure 1) and exported as a CSV file for subsequent analyses [1,7].

### 2.2. Environmental Data

A total of 29 environmental variables were selected (Table 1), including elevation (Ele), slope (Slo), aspect (Asp), normalized difference vegetation index (NDVI), food resource, human disturbance factors, climate variables (bio1–bio19), and land-use classification (LUCC). Current climate variables and elevation data were obtained from the WorldClim database (http://www.worldclim.org). Slope (Slo) and aspect (Asp) data were generated using the “3D Analyst” tool in ArcGIS. Food resources include distance to paddy field (DP), distance to major water (DW) and distance to beach (DB). Human disturbance factors consisted of distance to roads (DR) and distance to villages (DV). Land-use classification (LUCC) data were obtained from the Figshare platform (https://figshare.com), encompassing six categories—cropland, forest, grassland, bare land, water and town—under different climate scenarios for 2020. These data were imported into ArcGIS, and Euclidean distances were calculated to measure distances to paddy field, water source, beach, road, and village under various climate scenarios. NDVI data were obtained from the Resource and Environment Science and Data Center of the Chinese Academy of Sciences (https://www.gscloud.cn). Finally, all current variables were standardized with respect to boundaries, coordinate system (WGS_1984), and raster resolution (30”, approximately 1 km^2^), and the resulting data were exported in ASCII format [7].

Future bioclimatic variables for the White-naped Cranes correspond to the same 19 current climate variables. The 2050 period (2040–2060) and 2070 period (2060–2080) were selected as future time frames for analysis. The Shared Socioeconomic Pathways (SSPs) scenario framework, developed by the Intergovernmental Panel on Climate Change (IPCC) for climate change research, reflects the relationship between socioeconomic development patterns and climate change [22]. Three pathways—SSP126 (low emissions), SSP245 (medium emissions), and SSP585 (high emissions)—were employed to represent future CO_2_ emission concentration scenarios. By combining these pathways with the representative future periods of the 2050s and 2070s, six future climate models were ultimately defined: three models for the 2050s (2050s-SSP126, 2050s-SSP245, 2050s-SSP585) and three for the 2070s (2070s-SSP126, 2070s-SSP245, 2070s-SSP585). Terrain data (elevation, slope, aspect) were assumed to remain constant when projecting the future distribution of the White-naped Cranes. Land-use classification (LUCC) data from 2020 were applied to the current scenario, while future LUCC data corresponding to the 2050 and 2070 periods were obtained from WorldClim. To avoid model overfitting caused by autocorrelation and multicollinearity among environmental variables when predicting current potential suitable habitat, all environmental variables were imported into the MaxEnt model to obtain their contribution values. Based on correlation analysis between environmental variables using the sampling tool in ArcGIS 10.8 and SPSS 26 software, variables with smaller contribution values were removed from pairs of variables with a Spearman correlation coefficient r ≥ |0.8| [7].

### 2.3. Model Operation

The jackknife method was employed to assess the predictive capabilities of different environmental variables [23,24]. Variable response curves were generated to analyze the relationship between the probability of White-naped Crane presence and each variable. Twenty-five percent of White-naped Crane sites were randomly assigned as the test set for model validation, with the remaining 75% serving as the training set for model construction. Additionally, the “Write plot data” option was selected in the advanced settings to facilitate secondary plotting and statistical data analysis. For model output, the Subsample format was used, with the number of iterations set to 10 and all other parameters kept at default values. The final result was obtained as the average of the 10 modeling runs and output in “Logistic” format. Model accuracy was assessed using the area under the curve (AUC) of the receiver operating characteristic (ROC), with values ranging from 0 to 1: AUC < 0.6, no predictive value; 0.6 ≤ AUC < 0.7, weak predictive value; 0.7 ≤ AUC < 0.8, moderate predictive value; 0.8 ≤ AUC < 0.9, strong predictive value; 0.9 ≤ AUC ≤ 1, extremely strong predictive value [25].

### 2.4. Key Environmental Variables

Based on the contribution rates of environmental variables, replacement importance, and jackknife test results, a comprehensive analysis was conducted to identify the key environmental variables influencing habitat selection of the White-naped Cranes [26]. Environmental variables with higher contribution values indicate greater model reliance on those variables. Higher replacement importance suggests that the removal of a specific environmental variable results in a greater decline in the predictive power of the model, indicating a stronger influence of that variable on model outcomes [7]. The length variation of the “only this variable” and “excluding this variable” bands in the jackknife test results was used as an auxiliary criterion for identifying key environmental variables. A longer “only this variable” band indicates greater influence of that variable, whereas the “excluding this variable” band reflects the lower training score of the remaining variables’ total contribution after removal of a specific variable, indicating its greater impact [27]. Response curves for key environmental variables were obtained using the jackknife method. The range in which the probability exceeds 0.5 was defined as the suitable value range for that environmental variable [7,27].

### 2.5. Suitable Areas Classification

Maximum Training Sensitivity and Specificity (MTSS) precisely balances the risk of low sensitivity and low specificity [28,29]. Based on this criterion, the average MTSS value, obtained from ten MaxEnt model runs, was used as the threshold for suitable areas. Areas with distribution probability *p* ≤ MTSS were classified as unsuitable [3]. In this study, the average MTSS value was 0.09. The natural breakpoint method within the reclassification tool was used to divide suitable areas into three levels: high-suitable areas, mid-suitable areas, and low-suitable areas [30]. Specifically, unsuitable areas corresponded to 0 < *p* ≤ 0.1, low-suitable areas to 0.1 < *p* ≤ 0.2, mid-suitable areas to 0.2 < *p* ≤ 0.5, and high-suitable areas to 0.5 < *p* ≤ 1.0. The suitability classification method under future climate scenarios followed the same criteria.

Through field observations and analysis of occurrence data, the current occurrence points of the White-naped Crane were found not to occur in urban areas or regions above 600 m in elevation. Therefore, to remove high-altitude areas and urban areas from the potential suitable habitats, this study first used the Extract by Attributes tool to separately extract high, middle, and low suitable habitats for subsequent processing. Then, we used a sampling tool to extract the range of elevation values corresponding to the occurrence points of the White-naped Crane. Based on the elevation range of the occurrence data, areas with an elevation greater than 600 m were extracted using the Extract by Attributes tool. Finally, using the Erase tool from the Overlay toolset, the extracted elevation layer was overlaid with the high, middle, and low suitable habitat layers separately, and areas above 600 m in elevation were erased from each suitability class. Subsequently, we applied the Erase tool to remove urban areas from the high, middle, and low suitable habitats that had already been cleared of high-elevation areas.

### 2.6. Changes in the Suitable Areas and Centroid Shift

The “Quick Reclassify to Binary” tool in ArcGIS was used with *p* = 0.2 as the classification threshold: 0.2 < *p* ≤ 1.0 was defined as suitable habitat (high-suitable and mid-suitable areas), whereas 0 < *p* ≤ 0.2 was defined as unsuitable habitat (unsuitable and low-suitable areas). The “Distribution Changes Between Binary SDMs” toolkit within ArcGIS’s SDMToolbox was employed to analyze contraction and expansion areas of the White-naped Crane’s future wintering suitable habitat. Contraction areas were defined as regions where suitable habitat transitions to unsuitable habitat, whereas expansion areas were defined as regions where unsuitable habitat shifts to suitable habitat. Invariant areas corresponded to portions of the original suitable habitat that remained unchanged [10]. Finally, the “Centroid Changes” tool in ArcGIS 10.8 and BigMap software version 3.0 were employed to calculate the migration distances of the centroids of suitable habitat for the White-naped Cranes across different periods.

## 3. Results

### 3.1. Model Accuracy

**Figure 2:**
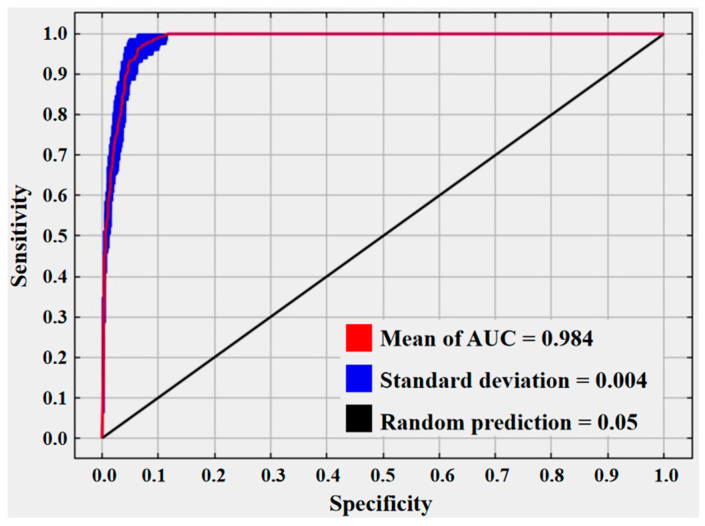
ROC curve of the MaxEnt model predicting the wintering distribution of the White-naped Cranes.

The mean AUC value of the MaxEnt model across 10 iterations was 0.984 ± 0.004 (mean ± SD), indicating high predictive accuracy and stability, with results reaching an excellent level (Figure 2).

### 3.2. Importance of Environment Variables

**Figure 3:**
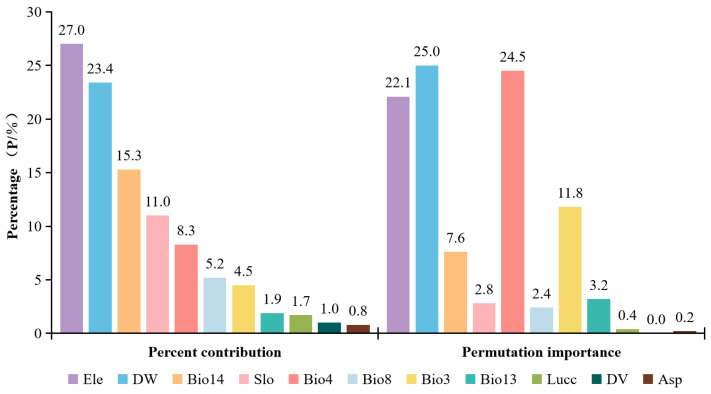
Contribution rates and replacement importance of 11 environmental variables affecting the wintering distribution of the White-naped Crane.

Model results indicated that among the 11 environmental variables included in the analysis, elevation (27.00%) contributed the most significantly to the wintering distribution of the White-naped Cranes. This was followed by distance to major water (23.40%), precipitation of the driest month (15.30%), slope (11.00%), temperature seasonality (8.30%), and mean temperature of the wettest quarter (5.20%). The environmental variable with the highest replacement importance was distance to major water (25.00%), followed by temperature seasonality (24.50%), elevation (22.10%), and isothermality (11.80%). Jackknife analysis revealed that, with only this variable considered, elevation scored highest in normalized training gain at 1.81, followed by distance to major water (1.39), isothermality (1.09), precipitation of the driest month (1.07), and slope (0.91). When modeling “excluding this variable,” the environmental variable with the greatest impact on model outcome variation was temperature seasonality, followed by distance to major water, precipitation of the driest month, and mean temperature of the wettest quarter (Figure 3). In summary, elevation, distance to major water, precipitation of the driest month, slope, temperature seasonality, and mean temperature of the wettest quarter were identified as the key environmental variables influencing the wintering distribution of the White-naped Cranes.

### 3.3. Key Environmental Variable Response Curves

**Figure 4:**
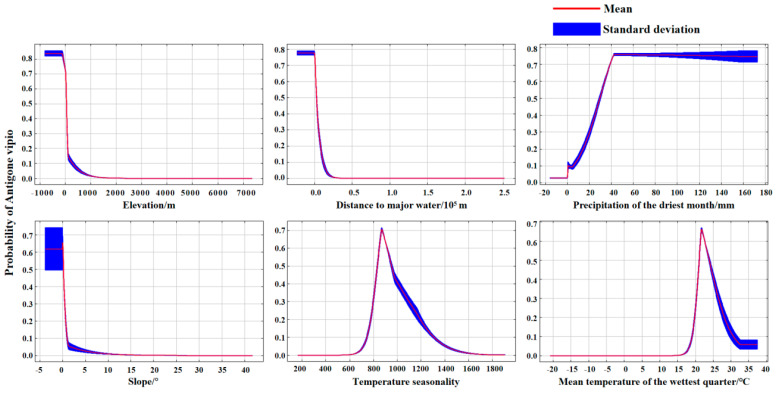
Response curves of the White-naped Crane to key environmental variables.

The probability of White-naped Crane presence exceeded 0.50 when elevation was less than 40.66 m and slope was less than 0.21°. Presence probability exceeded 0.50 when the distance to major water was less than 2.70 km. The probability of White-naped Crane occurrence exceeded 0.50 when precipitation of the driest month exceeded 29.70 mm, temperature seasonality ranged from 833.54 to 952.12, and mean temperature of the wettest quarter was between 21.08 and 23.88 °C (Figure 4).

### 3.4. Current Suitable Wintering Habitats for the White-Naped Cranes

**Figure 5:**
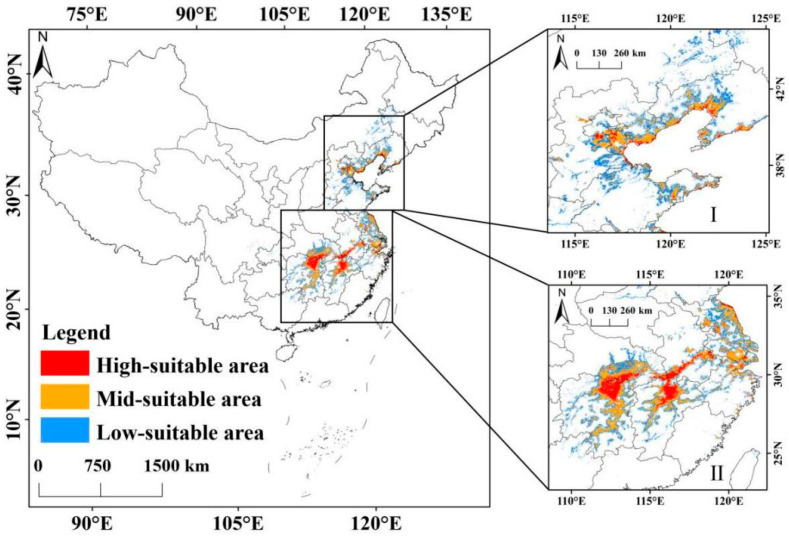
Distribution of current wintering habitats for the White-naped Crane: (**I**) Coastal areas of the Bohai Sea and the Yellow Sea; (**II**) Middle and lower reaches of the Yangtze River region.

Under the current climate pattern, suitable wintering areas for the White-naped Cranes were primarily distributed along the middle and lower reaches of the Yangtze River and along the Bohai and Yellow Seas. The high-suitable areas covered an area of 5.64 × 10^4^ km^2^, concentrated in parts of the middle and lower reaches of the Yangtze River, including Honghu Lake, Xiliang Lake, Futo Lake, Daguan Lake, Bohu Lake, and Wuchang Lake in Hubei Province; Dongting Lake in Hunan Province; Poyang Lake in Jiangxi Province; and Shengjin Lake in Anhui Province. The mid-suitable areas covered 16.71 × 10^4^ km^2^, extending around the high suitability zone. The low-suitable areas spanned 20.33 × 10^4^ km^2^, distributed not only around the middle and high suitability areas but also with scattered occurrences in locations such as Inner Mongolia, Chongqing, and Sichuan (Figure 5).

### 3.5. Suitable Habitats for the White-Naped Cranes and Land-Use Changes Under Future Climate Scenarios

**Figure 6:**
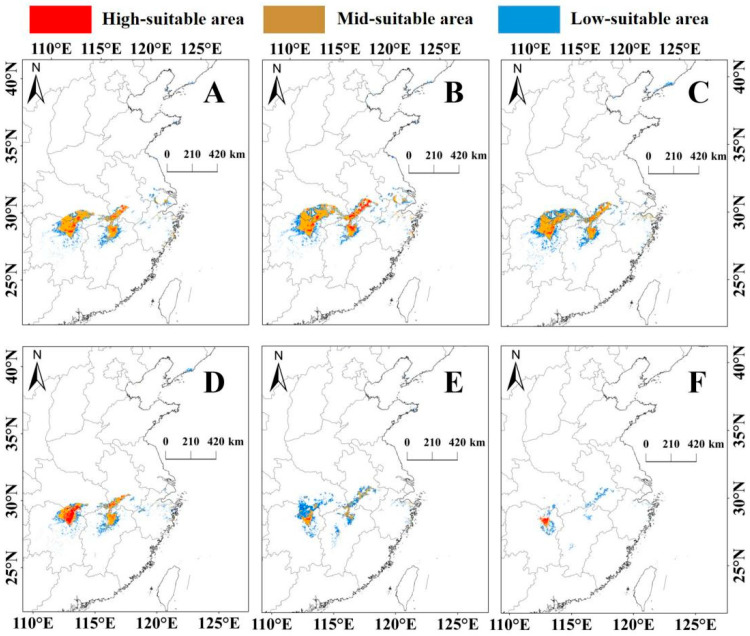
Distribution of wintering habitats for the White-naped Cranes under different future climate scenarios: (**A**) 2050s-SSP126; (**B**) 2050s-SSP245; (**C**) 2050s-SSP585; (**D**) 2070s-SSP126; (**E**) 2070s-SSP245; (**F**) 2070s-SSP585. The same below.

**Figure 7:**
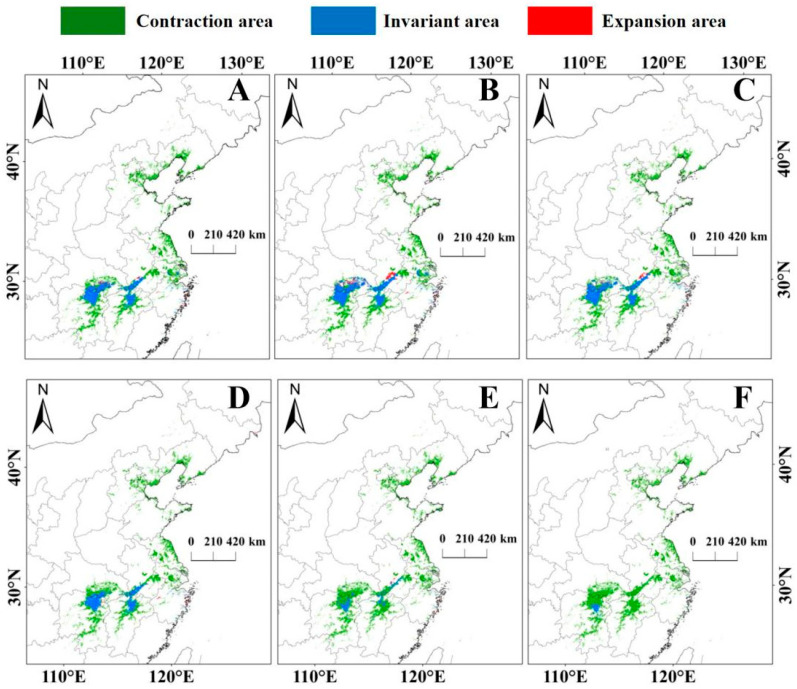
Changes in the distribution patterns of suitable habitats for the White-naped Cranes under different future climate scenarios: (**A**) 2050s-SSP126; (**B**) 2050s-SSP245; (**C**) 2050s-SSP585; (**D**) 2070s-SSP126; (**E**) 2070s-SSP245; (**F**) 2070s-SSP585. The same below.

The suitable habitat of the White-naped Cranes (high-suitable and mid-suitable areas) was projected to shrink significantly under future climate scenarios, being primarily concentrated in the middle and lower reaches of the Yangtze River. With increasing greenhouse gas emissions, suitable habitat was projected to gradually shift toward the middle reaches of the Yangtze River. Under future climate scenarios, suitable habitats were projected to contract primarily in Liaoning, Hebei, Tianjin, Shandong, Jiangsu, Zhejiang, Anhui, Jiangxi, Hunan, and Hubei. Invariant areas were projected to be concentrated in Hubei, Hunan, Anhui, and Jiangxi. Minimal expansions were projected in Hubei, Anhui, and Zhejiang (Figure 6). Under the three climate emission scenarios, the White-naped Crane’s suitable habitat was projected to contract most severely under the high-emission model (SSP585), followed by the medium-emission model (SSP245) and the low-emission model (SSP126) (Figure 7). Compared with current conditions, the smallest habitat contraction occurred under the 2050s-SSP245 model, shrinking from 22.35 × 10^4^ km^2^ to 6.31 × 10^4^ km^2^ (Table 2). The 2070s-SSP585 scenario exhibited the most severe contraction, shrinking from 22.35 × 10^4^ km^2^ to 0.56 × 10^4^ km^2^ (Table 2), with only a very small portion of suitable habitat remaining in parts of the middle Yangtze River region.

**Figure 8:**
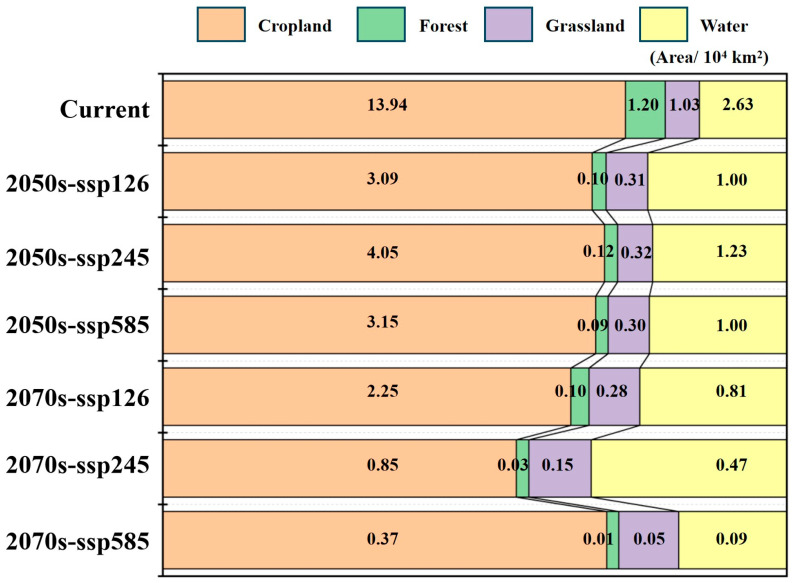
Changes in land-use classification of suitable habitats for the White-naped Crane under different climate scenarios.

In terms of land use, under various future scenarios, suitable habitats for the White-naped Crane were projected to undergo significant contraction, with all habitat types experiencing substantial area reductions. The most severe impacts were projected under the 2070s-SSP585 model. And the decline in cropland area was the most significant (Figure 8).

### 3.6. Changes in Key Environmental Variables Across Different Periods

In different future periods, contraction areas were projected to occur primarily in areas characterized by high elevation, long distance to major water, steep slopes, low precipitation in the driest month, large temperature seasonality, and high mean temperature in the wettest quarter. Within the invariant and expansion areas, environmental variables were projected to align more closely with the current suitability range for the White-naped Cranes (Table A1).

### 3.7. Distribution Centroid Shift of White-Naped Crane

**Figure 9:**
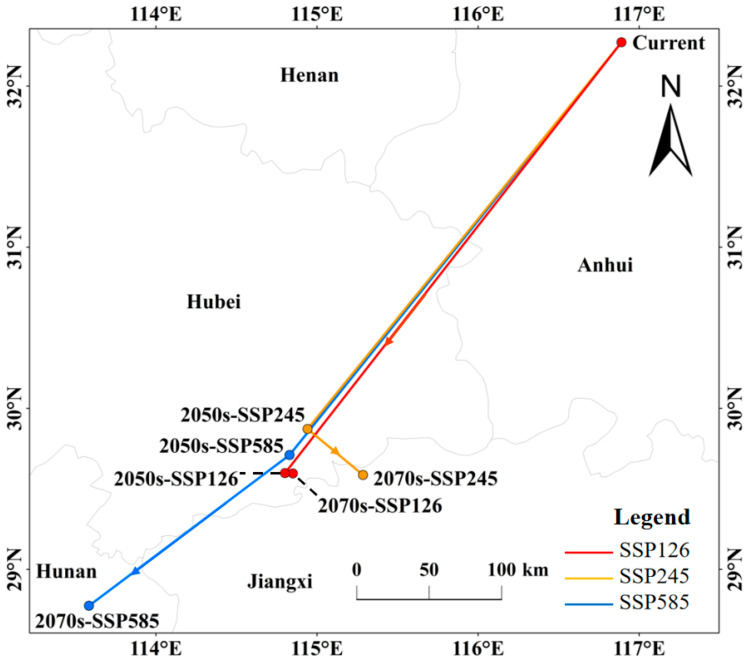
Centroid shift of suitable wintering habitats for the White-naped Cranes under different future climate scenarios.

The distribution centroid of the wintering habitat for the White-naped Cranes is currently located in Huainan City, Anhui Province (Table 3, Figure 9). Under future climate scenarios, the distribution centroid was projected to shift the farthest in linear distance under the SSP585 model, whereas the shifts under the SSP126 and SSP245 models were projected to be similar in linear distance (Figure 9). Over time, the distribution centroid under all three emission scenarios was projected to ultimately shift toward the southwest.

## 4. Discussion

### 4.1. Accuracy of the Maxent Model

The average AUC value across ten modeling iterations in this study was 0.984, indicating high predictive accuracy in identifying suitable wintering habitats of the White-naped Cranes [31,32]. A large sample size, precise mapping of distribution sites, and comprehensive selection of environmental variables enhance the predictive precision of the model [21,31,32]. Seventy-one valid sampling sites for the White-naped Cranes were obtained in this study, ensuring a sufficient sample size. Additionally, five categories of environmental variables were selected: 19 climatic factors, elevation, slope, land-use classification, and human disturbance factors. This comprehensive approach enhances the precision of the model’s simulated wintering distribution for the White-naped Cranes [32]. The current and future variables selected in this study adhere to the same construction framework, enabling accurate investigation of spatiotemporal changes in the potential suitable areas for the White-naped Cranes under future scenarios and their underlying drivers.

### 4.2. Key Environmental Variables and Causes

The key environmental variables influencing the wintering distribution of the White-naped Cranes were identified as topography (elevation and slope), water (distance to major water and precipitation of the driest month), and temperature (temperature seasonality and mean temperature of the wettest quarter).

Elevation was found to be the most significant environmental variable affecting the wintering distribution of the White-naped Cranes. The White-naped Crane prefers flat areas with elevations below 40.66 m and slopes less than 0.21°. Low-elevation and low-slope zones predominantly consist of farmlands, rivers, and lakes [33], which are rich in aquatic vegetation providing abundant food resources. Flat and open wetlands also facilitate predator avoidance [34]. Suitable temperatures in low-elevation zones play a crucial role in maintaining the White-naped Crane’s thermal balance [35]. As a typical wading bird, the White-naped Crane exhibits strong dependence on wetlands. The probability of White-naped Crane presence was found to increase with proximity to water sources in this study. The White-naped Crane primarily feeds on the tubers and seeds of aquatic plants, occasionally consuming animal-based foods, such as insects [36,37]. Wetland plants, commonly found near water sources in the middle and lower reaches of the Yangtze River, can provide the White-naped Crane with safe hiding places and abundant food resources [30,34]. Precipitation of the driest month affects the stability of water sources. Low rainfall during this period causes wetland desiccation and the death of aquatic plant roots and stems, resulting in a significant decline in the White-naped Crane’s food resources [38,39].

Temperature seasonality is defined as the degree of fluctuation in temperature over a given period [40]. High temperature seasonality reduces the stability of ambient temperatures, thereby affecting vegetation growth in wintering grounds (e.g., submerged plants) and ultimately influencing food availability for the White-naped Crane [41,42]. A stable environment is associated with reduced energy expenditure by White-naped Cranes during the wintering period [36,37]. Changes in mean temperature of the wettest quarter affect the dynamics of water bodies within the habitat [43]. Open aquatic habitats such as wetlands are preferred by the White-naped Crane for wintering [44]. The wettest season at Poyang Lake is summer, which is also the growing season for submerged plants. If temperature is too high during this season, evaporation increases, causing the water area to shrink. This results in a reduction in the area of suitable habitat for submerged plants, ultimately diminishing food resources for the White-naped Crane. In addition, temperature fluctuations in winter also have a significant impact on the White-naped Crane. If average temperatures are too low, water bodies may freeze, thereby hindering foraging [44,45,46]. Conversely, if temperatures are too high, increased evaporation may significantly reduce the extent of suitable wintering habitat [44,45,46].

Overall, during the wintering period, the White-naped Crane is strongly associated with wetlands characterized by low elevation, gentle slopes, proximity to water sources, and low temperature variability, whereas areas with high levels of human activity are generally avoided. The areas of high habitat suitability identified in this study, such as Poyang Lake and Dongting Lake, are likely to provide these environmental conditions, as they are characterized by a relatively mild climate, abundant water and food resources, and suitable and secure foraging and roosting habitats. However, empirically, the White-naped Crane has not been documented in all areas that meet these suitability criteria. This discrepancy may be explained by the species’ migratory behavior, as its migration routes are largely confined to wetland systems in eastern China. Additionally, geographical barriers and flight energy costs are likely to further constrain the accessibility of these regions to the species.

### 4.3. Current Suitable Wintering Habitats for the White-Naped Cranes

Currently, suitable wintering habitats for the White-naped Cranes are primarily distributed in wetlands along the middle and lower reaches of the Yangtze River, as well as along the coasts of the Yellow and Bohai Seas. The middle and lower reaches of the Yangtze River exhibit a mild winter climate and contain large freshwater lakes, including Dongting Lake, Poyang Lake, and Shengjin Lake. Multiple national nature reserves have been established in these regions, which are characterized by relatively low levels of human disturbance. Surrounding farmlands also provide consistent food resources for the species. Land-use classification analyses indicate that cropland accounts for a substantial proportion of suitable habitat under both current and projected climate scenarios. Consequently, in periods of drought when food availability in highly suitable lakes and wetlands is reduced, artificial paddy fields become critical foraging grounds for the species. Field surveys conducted at Poyang Lake indicate that both the White-naped Crane and other crane species utilize post-harvest rice fields extensively for foraging [18]. Thus, the combination of abundant food resources and favorable climatic conditions in the middle and lower reaches of the Yangtze River attracts White-naped Cranes to overwinter in this region [46,47]. Similarly, wetlands along the Bohai and Yellow Seas contain abundant aquatic plant tubers, open wetland landscapes, and relatively mild climates that fulfill the ecological requirements for White-naped Cranes [44,46].

The northernmost suitable wintering habitat for the White-naped Cranes in China (high-suitable and mid-suitable areas) is situated along the Bohai Sea coast of Liaoning Province (Table A1), similar to the northernmost wintering limits of the Siberian Crane and the Hooded Crane [6,7,21]. Low winter temperatures in high-latitude regions are likely the primary factor restricting the northern expansion of the White-naped Crane [44,45,46]. The Common Crane (*Grus grus*) is able to persist in more northern regions, such as Heilongjiang Province [31], owing to its greater environmental adaptability and capacity to utilize arid habitats, including farmland, whereas the White-naped Crane is more dependent on natural wetlands [37,44,45].

### 4.4. Spatiotemporal Changes in Suitable Habitats for the White-Naped Cranes Under Future Climate Scenarios

Under different future climate scenarios, the suitable wintering habitat of the White-naped Cranes is projected to gradually contract toward the middle reaches of the Yangtze River, displaying a spatial pattern characterized by substantial contraction with limited expansion. By 2050, under all three climate scenarios, the suitable habitat for the White-naped Cranes is projected to significantly shrink in northern regions, remain relatively stable in the middle and lower reaches of the Yangtze River, and exhibit minor expansion in certain areas compared to the present. By 2070, suitable habitats for the White-naped Cranes are projected to almost disappear in northern regions, gradually concentrating in parts of the middle Yangtze River basin, with minimal expansion in Anhui and Zhejiang provinces. The results indicate that the contraction areas are characterized by high elevation, long distance to major water bodies, steep slopes, low precipitation of the driest month, high temperature seasonality, and elevated mean temperature in the wettest quarter (Table A1). This pattern is attributed to the fact that water resources available to the White-naped Cranes in high-altitude areas are significantly less abundant than in the low-altitude regions of the middle and lower Yangtze River [48,49,50]. Rising greenhouse gas concentrations elevate temperatures, further diminishing water resources in high-altitude areas [51,52]. Significant depletion of water resources increases the distance to major water, while precipitation of the driest month gradually declines owing to reduced water availability [53,54,55]. Furthermore, rising temperatures elevate mean temperature of the wettest quarter (typically in summer) leading to reductions in water body size and ultimately affecting winter water availability [53,54,55,56]. The White-naped Crane exhibits high sensitivity to these critical factors, preferring low-elevation, temperate regions with relatively abundant water resources and sufficient winter precipitation (Figure 4). Consequently, over time, increasing greenhouse gas emissions are projected to render high-elevation northern areas progressively unsuitable for the species. Under projected future greenhouse gas scenarios, White-naped Cranes are expected to concentrate in the lower-elevation regions of the middle and lower Yangtze River.

Currently, the wintering ranges of the White-naped Cranes are broadly similar to those of the Siberian Cranes and the Hooded Cranes. Under future climate scenarios, the wintering ranges of the Siberian Cranes and the Hooded Cranes are projected to shift northward, while suitable habitats in the middle and lower Yangtze River are projected to decline significantly [6,7]. This pattern is primarily driven by minimum temperature during the coldest season, which strongly influence the wintering distributions of these species. With increasing greenhouse gas emissions, water bodies in some northern regions are more likely to remain unfrozen during the coldest season, thereby expanding suitable habitat [6,7]. This change may shorten migration distances between breeding and wintering grounds, thereby reducing energy expenditure and migration risk [6,7]. In contrast, the wintering distribution of the White-naped Cranes is strongly influenced by elevation, distance to major water, and precipitation of the driest month. Under climate warming, its wintering distribution has become increasingly concentrated in parts of the middle Yangtze River region, accompanied by a marked reduction in suitable habitat. The contraction of suitable habitat in China indicates a substantial reduction in available living space for the species. Further investigation is required to assess whether the remaining habitat is sufficient to support the current population and to evaluate its implications for winter survival. Notably, White-naped Crane populations wintering in Japan and South Korea have shown signs of recovery, likely due to artificial food supplementation [35]. In the future, the impacts of climate change and natural wetland restoration on the population size and distribution of the White-naped Crane in China should be promptly assessed, and appropriate conservation measures should be taken accordingly.

### 4.5. Future Shift in the Distribution Centroid for the White-Naped Cranes

Under projected future climate scenarios, the distribution centroid of the White-naped Cranes exhibits an overall southwestward shift. By 2050, the wintering centroid is projected to migrate 320–360 km southwestward under all three emission scenarios. By 2070, under SSP126 and SSP245 scenarios, the migration distance is projected to be less than 50 km, whereas under SSP585, the centroid is projected to continue shifting southwestward to Yueyang, Hunan province, with a migration distance substantially exceeding that of the other two scenarios. This pattern indicates that: (1) over time (2050–2070), the magnitude of centroid shifts diminishes; (2) with increasing greenhouse gas emissions, the middle reaches of the Yangtze River are projected to become the primary wintering habitat for the White-naped Cranes, while the lower reaches are projected to provide extremely limited wintering resources. The shift in the White-naped Crane’s centroid contrasts with the northeastward migration observed for the Hooded Cranes and Siberian Cranes [6,7]. The increase in minimum temperature during the coldest season is identified as the primary driver of the northeastward shift of wintering centroid for the Hooded Cranes and Siberian Cranes. In contrast, the wintering pattern of the White-naped Cranes appears less influenced by temperature changes during the coldest season. This study demonstrates that elevation and distance to major water exert the strongest influence on wintering behavior, with the species showing a preference for areas with stable precipitation. Northern regions are characterized by relatively scarce water resources (Table A1). Projected changes in elevation and distance to major water in the middle Yangtze River region are expected to be more conducive to habitat suitability for the White-naped Cranes (Figure 6, Table A1).

### 4.6. Conservation Recommendations

In MaxEnt model predictions, the suitable wintering habitat of the White-naped Cranes is projected to exhibit a gradual decline, with a distribution shift toward the middle reaches of the Yangtze River. The distribution is strongly influenced by key environmental variables, including elevation, distance to major water, precipitation of the driest month, slope, temperature seasonality, and mean temperature of the wettest quarter. Based on these findings, the following conservation recommendations are proposed for the White-naped Cranes:

(1) Optimize nature reserve development: With the projected shift of wintering habitats toward the middle reaches of the Yangtze River, priority should be given to the Dongting Lake region in Hunan Province. Although multiple national and provincial nature reserves have been established around Dongting Lake, wetland fragmentation remains within these protected areas. Measures such as ditch restoration, and the construction of ecological corridors should be implemented to rehabilitate wetlands.
(2) Strengthen hydrological monitoring: Given that distance to major water and precipitation of the driest month significantly influence suitable wintering habitats, monitoring of hydrological changes should be intensified. Fluctuations in water levels affect the growth of submerged vegetation, thereby impacting crane foraging. Therefore, rational management of water level fluctuations is essential.
(3) Scientific assessment of population dynamics and suitable habitat conditions: Under projected future climate scenarios, existing suitable habitats in inland lakes such as Poyang Lake (Jiangxi) and Caizi Lake (Anhui) are at risk of loss. Scientific evaluations of population dynamics and habitat suitability in these regions should be conducted, including assessments of age ratios, food availability, and changes in habitat quality. Protective measures should be implemented prior to the loss of suitable habitats.
(4) Reduce human disturbance: The quality of natural suitable habitats for the White-naped Cranes has declined because of the increased human interference. Such interference affects crane foraging, resting, and vigilance behaviors. Enhanced management of restricted access zones, limitations on human activities, and reduction in disturbances are expected to improve the quality of natural suitable habitats. Furthermore, human activities contribute to climate warming, which significantly impacts wetlands. Adoption of low-emission strategies is recommended to protect suitable habitats for waterbirds.

## 5. Conclusions

The MaxEnt model was applied to simulate the distribution and spatial patterns of suitable wintering habitats for the White-naped Cranes under current and future climate scenarios. The results indicate that current suitable wintering habitats are primarily distributed along the middle and lower Yangtze River and the Bohai and Yellow Sea coasts. Under future climate scenarios, suitable wintering habitats are projected to decline significantly, and the distribution centroid is projected to shift southwestward. Elevation, distance to major water, and precipitation of the driest month are identified as key environmental factors driving habitat contraction. Habitat contraction intensifies over time and with increasing greenhouse gas emissions; the SSP585 scenario shows the greatest shift in distribution centroid and the most severe contraction. One limitation of this study is the lack of consideration of key ecological factors, including flock size, food resources, interspecific interactions, migration dynamics, environmental carrying capacity, and minimum suitable habitat area, which may introduce bias into the prediction of future wintering habitat suitability for the White-naped Crane. Consequently, it cannot be determined whether the projected extent of suitable habitat is sufficient to support the survival of the White-naped Crane. Future research should include a comprehensive assessment of habitat suitability for this species by integrating these ecological variables across multiple spatial and temporal scales. Nevertheless, the wintering distribution of the White-naped Cranes was simulated under future climate and land-use scenarios, and the key environmental drivers and their projected trends were systematically analyzed.

## Figures and Tables

**Table 1:** Environment variables and their descriptions.

Variable	Description	Unit
bio 1	Annual mean temperature	°C
bio 2	Mean diurnal temperature range	°C
bio 3	Isothermality	—
bio 4	Temperature seasonality	—
bio 5	Max temperature of the warmest month	°C
bio 6	Min temperature of the coldest month	°C
bio 7	Temperature annual range	°C
bio 8	Mean temperature of the wettest quarter	°C
bio 9	Mean temperature of the driest quarter	°C
bio 10	Mean temperature of the warmest quarter	°C
bio 11	Mean temperature of the coldest quarter	°C
bio 12	Annual precipitation	mm
bio 13	Precipitation of the wettest month	mm
bio 14	Precipitation of the driest month	mm
bio 15	Precipitation seasonality (coefficient of variation)	—
bio 16	Precipitation of the wettest quarter	mm
bio 17	Precipitation of the driest quarter	mm
bio 18	Precipitation of the warmest quarter	mm
bio 19	Precipitation of the coldest quarter	mm
Ele	Elevation	m
Asp	Aspect	—
Slo	Slope	°
NDVI	Normalized difference vegetation index	—
LUCC	Land-use classification	—
DP	Distance to paddy field	m
DW	Distance to major water	m
DB	Distance to beach	m
DR	Distance to road	m
DV	Distance to village	m

**Table 2:** Areas of high, middle, and low Suitable areas for the wintering White-naped Cranes under different climate scenarios.

Climatic Mode	High-Suitable Area (km^2^)	Mid-Suitable Area (km^2^)	Low-Suitable Area (km^2^)
Current	5.64 × 10^4^	16.71 × 10^4^	20.33 × 10^4^
2050s-SSP126	0.57 × 10^4^	4.44 × 10^4^	3.03 × 10^4^
2050s-SSP245	0.94 × 10^4^	5.37 × 10^4^	3.97 × 10^4^
2050s-SSP585	0.30 × 10^4^	4.73 × 10^4^	4.01 × 10^4^
2070s-SSP126	1.06 × 10^4^	2.76 × 10^4^	2.38 × 10^4^
2070s-SSP245	0.08 × 10^4^	1.57 × 10^4^	3.50 × 10^4^
2070s-SSP585	0.22 × 10^4^	0.34 × 10^4^	1.01 × 10^4^

**Table 3:** Changes in the area and centroid of suitable wintering habitats for the White-naped Cranes under different future climate and land-use scenarios.

Climatic Mode	Area (km^2^)			Centroid Coordinate	Migration Distance (km)
	Expansion Area	Invariant Area	Contraction Area		
current				116.89° E, 32.27° N	
2050s-SSP126	0.27 × 10^4^	4.73 × 10^4^	15.17 × 10^4^	114.80° E, 29.60° N	357.49
2050s-SSP245	0.77 × 10^4^	5.47 × 10^4^	14.83 × 10^4^	114.94° E, 29.87° N	324.69
2050s-SSP585	0.39 × 10^4^	4.62 × 10^4^	14.89 × 10^4^	114.83° E, 29.71° N	345.58
2070s-SSP126	0.15 × 10^4^	3.67 × 10^4^	14.94 × 10^4^	114.85° E, 29.60° N	4.79
2070s-SSP245	0.10 × 10^4^	1.55 × 10^4^	17.36 × 10^4^	115.29° E, 29.59° N	45.97
2070s-SSP585	0.03 × 10^4^	0.53 × 10^4^	17.36 × 10^4^	113.58° E, 28.77° N	159.43

## Data Availability

The data presented in this study are available upon request from the corresponding author. The data are not publicly available due to the constraint in the consent.

## Appendix A

**Table A1:** Changes in key environmental variables under different modes.

Period	Region	Climate Scenarios	Average Key Environmental Variables					
			Bio14/mm	Bio4	Bio8/°C	Ele/m	DW/m	Slo/°
Current	High-suitable area		34.22	910.02	22.47	21.04	2076.99	0.12
2050s	Contraction area	SSP126	27.90	975.67	27.49	52.24	7794.18	0.32
		SSP245	32.42	979.78	27.63	51.29	7904.84	0.35
		SSP585	29.55	981.62	28.61	49.95	7539.25	0.35
	Invariant area	SSP126	43.10	896.52	24.39	23.37	1890.57	0.14
		SSP245	48.65	914.69	24.97	23.52	1868.92	0.14
		SSP585	43.29	926.49	25.77	23.94	1778.16	0.13
	Expansion area	SSP126	37.30	874.81	23.73	33.86	3699.16	0.41
		SSP245	36.30	918.89	24.19	30.13	4134.82	0.32
		SSP585	34.53	916.79	25.03	30.07	3596.64	0.29
2070s	Contraction area	SSP126	31.37	963.96	28.34	50.69	6975.55	0.35
		SSP245	26.71	958.89	27.61	46.09	6619.88	0.31
		SSP585	32.83	982.65	29.37	44.55	6113.57	0.30
	Invariant area	SSP126	47.49	896.62	23.36	25.17	1799.65	0.16
		SSP245	37.98	909.58	23.47	21.34	1351.66	0.15
		SSP585	54.57	933.61	22.93	35.30	2334.04	0.19
	Expansion area	SSP126	37.84	878.29	22.96	72.80	3319.72	1.08
		SSP245	29.79	866.90	24.42	15.03	3802.36	0.46
		SSP585	48.25	896.24	23.56	63.01	4667.27	0.55
